# (2*E*)-*N*′-Benzoyl-3-(4-nitro­phen­yl)prop-2-enohydrazide

**DOI:** 10.1107/S1600536809053379

**Published:** 2009-12-16

**Authors:** Samir A. Carvalho, Edson F. da Silva, Marcus V. N. de Souza, Edward R. T. Tiekink, James L. Wardell, Solange M. S. V. Wardell

**Affiliations:** a Instituto de Química, Universidade Federal do Rio de Janeiro, 21949-900 Rio de Janeiro, RJ, Brazil; b Departamento de Síntese Orgánica, Instituto de Tecnologia em Fármacos FIOCRUZ, Manguinhos, Rua Sizenando Nabuco 100, Manguinhos, 21041-250 Rio de Janeiro, RJ, Brazil; c Department of Chemistry, University of Malaya, 50603 Kuala Lumpur, Malaysia; d Centro de Desenvolvimento Tecnológico em Saúde (CDTS), Fundação Oswaldo Cruz (FIOCRUZ), Casa Amarela, Campus de Manguinhos, Av. Brasil 4365, 21040-900 Rio de Janeiro, RJ, Brazil; e CHEMSOL, 1 Harcourt Road, Aberdeen AB15 5NY, Scotland

## Abstract

In the title compound, C_16_H_13_N_3_O_4_, the dihedral angle between the terminal benzene rings is 14.02 (7)°. The carbonyl groups are *anti* with respect to each other, which facilitates their participation in the formation of supra­molecular chains. Each side of the –C(=O)N(H)N(H)C(=O)– residue associates with a centrosymmetrically related mol­ecule, resulting in the formation of essentially flat ten-membered {⋯O=CNN(H)}_2_ synthons. The resultant chains are further consolidated in the crystal structure *via* C—H⋯O contacts.

## Related literature

For background to the biological activity of *trans*-cinnamic acid derivatives, see: Bezerra *et al.* (2006 ▶); Chung & Shin (2007 ▶); Naz *et al.* (2006 ▶). For background to the development of hydrazide derivatives for biological evaluation, see: Carvalho *et al.* (2008 ▶, 2009 ▶).
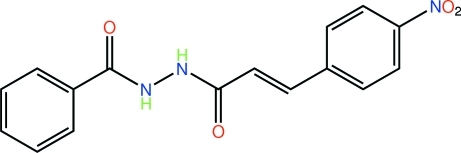


## Experimental

### 

#### Crystal data

C_16_H_13_N_3_O_4_
*M* *_r_* = 311.29
Triclinic, 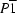

*a* = 6.8263 (2) Å
*b* = 9.6483 (3) Å
*c* = 10.8571 (3) Å
α = 95.535 (2)°
β = 102.701 (2)°
γ = 91.728 (2)°
*V* = 693.35 (4) Å^3^
*Z* = 2
Mo *K*α radiation
μ = 0.11 mm^−1^
*T* = 120 K
0.50 × 0.40 × 0.20 mm

#### Data collection

Nonius KappaCCD diffractometer
Absorption correction: multi-scan (*SADABS*; Sheldrick, 2007 ▶) *T*_min_ = 0.664, *T*_max_ = 0.746
15199 measured reflections
3157 independent reflections
2495 reflections with *I* > 2σ(*I*)
*R*_int_ = 0.047

#### Refinement

*R*[*F* ^2^ > 2σ(*F* ^2^)] = 0.043
*wR*(*F* ^2^) = 0.127
*S* = 1.06
3157 reflections
214 parameters
2 restraints
H atoms treated by a mixture of independent and constrained refinement
Δρ_max_ = 0.31 e Å^−3^
Δρ_min_ = −0.31 e Å^−3^

### 

Data collection: *COLLECT* (Hooft, 1998 ▶); cell refinement: *DENZO* (Otwinowski & Minor, 1997 ▶) and *COLLECT*; data reduction: *DENZO* and *COLLECT*; program(s) used to solve structure: *SHELXS97* (Sheldrick, 2008 ▶); program(s) used to refine structure: *SHELXL97* (Sheldrick, 2008 ▶); molecular graphics: *ORTEP-3* (Farrugia, 1997 ▶) and *DIAMOND* (Brandenburg, 2006 ▶); software used to prepare material for publication: *publCIF* (Westrip, 2009 ▶).

## Supplementary Material

Crystal structure: contains datablocks global, I. DOI: 10.1107/S1600536809053379/hb5278sup1.cif

Structure factors: contains datablocks I. DOI: 10.1107/S1600536809053379/hb5278Isup2.hkl

Additional supplementary materials:  crystallographic information; 3D view; checkCIF report

## Figures and Tables

**Table 1:** Hydrogen-bond geometry (Å, °)

*D*—H⋯*A*	*D*—H	H⋯*A*	*D*⋯*A*	*D*—H⋯*A*
N2—H2n⋯O4	0.88 (1)	2.27 (1)	2.6470 (16)	106 (1)
N2—H2n⋯O4^i^	0.88 (1)	2.05 (1)	2.8721 (15)	155 (1)
N3—H3n⋯O3	0.88 (1)	2.35 (1)	2.6687 (15)	101 (1)
N3—H3n⋯O3^ii^	0.88 (1)	2.07 (1)	2.9269 (15)	165 (1)
C12—H12⋯O4^iii^	0.95	2.56	3.4257 (18)	151
C14—H14⋯O1^iv^	0.95	2.58	3.2851 (19)	132

Symmetry codes: (i) 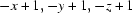; (ii) 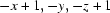; (iii) 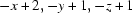; (iv) 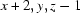.

**Fig. 1.**
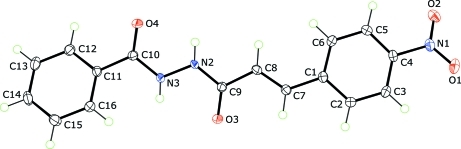
The molecular structure of (I) showing displacement ellipsoids at the 50% probability level.

**Fig. 2.**
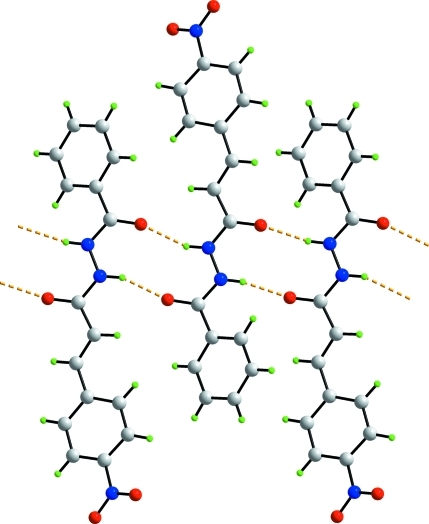
A view of the supramolecular chain in (I) mediated by N–H···O hydrogen bonding (orange dashed lines). Colour code: O, red; N, blue; C, grey; and H, green.

**Fig. 3.**
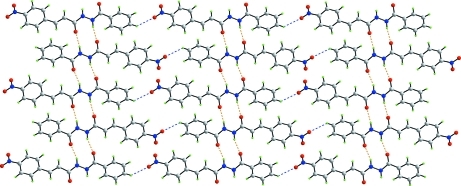
A view of the supramolecular array in (I) with N–H···O hydrogen bonding and C–H···N contacts shown as orange and blue dashed lines, respectively. Colour code: O, red; N, blue; C, grey; and H, green.

**Fig. 4.**
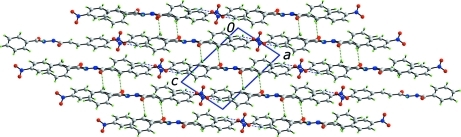
A view of the stacking of layers (illustrated in Fig. 3) in (I) with the C—H···O contacts shown as green dashed lines. Colour code: O, red; N, blue; C, grey; and H, green.

## supplementary crystallographic information

### Comment

Tuberculosis (TB) remains among the world's great public health challenges. Worldwide resurgence of TB is due to two major problems: the AIDS epidemic, which started in the mid-1980 s, and the outbreak of multi-drug resistant (MDR) TB (Bezerra *et al.*, 2006; Chung & Shin 2007; Naz *et al.*, 2006). In connection with on-going studies designed to generate novel therapeutic anti-malarial agents, we recently described a new class of isonicotinic and benzoic acid *N*'-(3-phenyl-acryloyl)-hydrazide derivatives as attractive anti-tubercular agents (Carvalho *et al.*, 2008). Allied with these investigations are structural studies: the structure of *N*'-[(2*E*)-3-phenylprop-2-enoyl]benzohydrazide was recently reported by us (Carvalho *et al.*, 2009) and now we report the structure of title compound, (I).

The molecular structure of (I), Fig. 1, shows small but significant deviations from co-planarity. Thus, the central moiety is essentially planar as seen in the sequence of C1–C7–C8–C9, C7–C8–C9–N2 and C9–N2–N3–C10 torsion angles of 175.50 (12), 173.72 (12) and 172.40 (12) °, respectively. However, the terminal amide-bound benzene ring is significantly twisted out of the plane of the remaining molecule: the N3–C10–C11–C12 torsion angle = -153.50 (13) °. This contrasts the co-planarity of the nitro-substituted benzene ring: the C2–C1–C7–C8 torsion angle = -179.69 (13) °. However the nitro group is twisted out of the plane of the benzene ring to which it is attached: the O1–N1–C4–C3 torsion angle is -17.9 (2) °. The overall twist in the molecule is reflected in the dihedral angle formed between the benzene rings of 14.02 (7) °. The conformation about the C7═C8 bond [1.3360 (19) Å] is *E*. The carbonyl groups are *anti* with respect to each other, a conformation that allows their participation in the stabilization of supramolecular chains. Each side of the –C(═O)N(H)N(H)C(═O)- residue associates with a centrosymmetrically related molecule resulting in the formation of essentially flat ten-membered {···O═CNN(H)}_2_ synthons, Fig. 2 and Table 1. The overall topology of the chain orientation along the *b* axis is flat. The N–H···O hydrogen bonds are not linear as might be expected owing to the presence of weaker intramolecular N–H···O contacts, Table 1. Supramolecular chains are connected into a layer motif *via* C_phenyl_–*H*···O_nitro_ contacts, Fig. 3 and Table 1. It is assumed that the twist of the nitro group from the plane of the benzene ring (see above) arises to optimize this contact. These supramolecular arrays are linked into the three-dimensional structure *via* C_phenyl_–*H*···O_carbonyl_ interactions, Fig. 4 and Table 1.

### Experimental

4-Nitrophenyl (2*E*)-3-(4-nitrophenyl)-2-propenoate (2 g), prepared by successive treatments of *trans*-4-nitrocinnamic acid with thionyl chloride and 4-nitrophenol, was added to a solution of PhCONHNH_2_ (1.1 equiv.) in pyridine (40 ml). After refluxing the reaction mixture for 6 h, the pyridine was removed under vacuum and H_2_O (20 ml) was added. The precipitate was collected, washed with H_2_O (yield 80%) and recrystallized from EtOH to yield orange blocks of (I), m.pt. 551–552 K. ^1^H NMR (500.00 MHz, DMSO-d_6_) δ: 7.00 (1*H*, d, J = 16.0 Hz), 7.61 (3*H*, m), 7.74 (1*H*, d, J = 16.0 Hz), 7.96 (2*H*, d, J = 7.5 Hz), 7.99 (2*H*, d, J = 8.5 Hz), 8.36 (2*H*, d, J = 8.5 Hz), 10.55 (1*H*, s, NH), 10.71 (1*H*, s, NH) p.p.m.. ^13^C NMR (125 MHz, DMSO-d_6_) δ: 166.02, 164.06, 147.67, 140.73, 138.35, 132.09, 131.88, 128.81, 128.54, 127.30, 123.99, 123.14 p.p.m.

### Refinement

The C-bound H atoms were geometrically placed (C–H = 0.95 Å) and refined as riding with *U_iso_*(H) = 1.2*U_eq_*(C). The N–H atoms were located in a difference map and refined with the distance restraint N–H = 0.88±0.01 and with *U_iso_*(H) = 1.2*U_eq_*(N).

### Crystal data

**Table:**

C_16_H_13_N_3_O_4_	*Z* = 2
*M**_r_* = 311.29	*F*(000) = 324
Triclinic, *P*1	*D*_x_ = 1.491 Mg m^−^^3^
Hall symbol: -P 1	Mo *K*α radiation, λ = 0.71073 Å
*a* = 6.8263 (2) Å	Cell parameters from 7821 reflections
*b* = 9.6483 (3) Å	θ = 2.9–27.5°
*c* = 10.8571 (3) Å	µ = 0.11 mm^−^^1^
α = 95.535 (2)°	*T* = 120 K
β = 102.701 (2)°	Block, orange
γ = 91.728 (2)°	0.50 × 0.40 × 0.20 mm
*V* = 693.35 (4) Å^3^	

### Data collection

**Table:**

Nonius KappaCCD diffractometer	3157 independent reflections
Radiation source: Enraf Nonius FR591 rotating anode	2495 reflections with *I* > 2σ(*I*)
10 cm confocal mirrors	*R*_int_ = 0.047
Detector resolution: 9.091 pixels mm^-1^	θ_max_ = 27.5°, θ_min_ = 3.0°
φ and ω scans	*h* = −8→8
Absorption correction: multi-scan (*SADABS*; Sheldrick, 2007)	*k* = −12→12
*T*_min_ = 0.664, *T*_max_ = 0.746	*l* = −14→13
15199 measured reflections	

### Refinement

**Table:**

Refinement on *F*^2^	Primary atom site location: structure-invariant direct methods
Least-squares matrix: full	Secondary atom site location: difference Fourier map
*R*[*F*^2^ > 2σ(*F*^2^)] = 0.043	Hydrogen site location: inferred from neighbouring sites
*wR*(*F*^2^) = 0.127	H atoms treated by a mixture of independent and constrained refinement
*S* = 1.06	*w* = 1/[σ^2^(*F*_o_^2^) + (0.0687*P*)^2^ + 0.1871*P*] where *P* = (*F*_o_^2^ + 2*F*_c_^2^)/3
3157 reflections	(Δ/σ)_max_ < 0.001
214 parameters	Δρ_max_ = 0.31 e Å^−^^3^
2 restraints	Δρ_min_ = −0.31 e Å^−^^3^

### Special details

**Table:**

Geometry. All s.u.'s (except the s.u. in the dihedral angle between two l.s. planes) are estimated using the full covariance matrix. The cell s.u.'s are taken into account individually in the estimation of s.u.'s in distances, angles and torsion angles; correlations between s.u.'s in cell parameters are only used when they are defined by crystal symmetry. An approximate (isotropic) treatment of cell s.u.'s is used for estimating s.u.'s involving l.s. planes.
Refinement. Refinement of *F*^2^ against ALL reflections. The weighted *R*-factor *wR* and goodness of fit *S* are based on *F*^2^, conventional *R*-factors *R* are based on *F*, with *F* set to zero for negative *F*^2^. The threshold expression of *F*^2^ > 2σ(*F*^2^) is used only for calculating *R*-factors(gt) *etc*. and is not relevant to the choice of reflections for refinement. *R*-factors based on *F*^2^ are statistically about twice as large as those based on *F*, and *R*- factors based on ALL data will be even larger.

### Fractional atomic coordinates and isotropic or equivalent isotropic displacement parameters (Å^2^)

**Table:**

	*x*	*y*	*z*	*U*_iso_*/*U*_eq_	
O1	−0.61174 (17)	0.25925 (13)	1.01283 (12)	0.0338 (3)	
O2	−0.39300 (16)	0.42685 (11)	1.10137 (10)	0.0231 (3)	
O3	0.30141 (14)	0.06073 (10)	0.52732 (10)	0.0185 (2)	
O4	0.68693 (15)	0.44415 (10)	0.44917 (10)	0.0214 (3)	
N1	−0.45702 (18)	0.33067 (13)	1.01817 (12)	0.0203 (3)	
N2	0.44535 (17)	0.27625 (12)	0.53463 (11)	0.0154 (3)	
H2N	0.444 (2)	0.3677 (10)	0.5437 (15)	0.018*	
N3	0.56991 (17)	0.22492 (12)	0.45712 (11)	0.0155 (3)	
H3N	0.585 (2)	0.1348 (10)	0.4560 (15)	0.019*	
C1	−0.1035 (2)	0.22612 (15)	0.75127 (13)	0.0150 (3)	
C2	−0.2558 (2)	0.13309 (15)	0.76568 (13)	0.0180 (3)	
H2	−0.2777	0.0448	0.7166	0.022*	
C3	−0.3753 (2)	0.16829 (16)	0.85096 (14)	0.0204 (3)	
H3	−0.4784	0.1048	0.8610	0.024*	
C4	−0.3414 (2)	0.29731 (15)	0.92086 (13)	0.0169 (3)	
C5	−0.1974 (2)	0.39458 (15)	0.90566 (14)	0.0191 (3)	
H5	−0.1808	0.4843	0.9523	0.023*	
C6	−0.0783 (2)	0.35802 (15)	0.82096 (14)	0.0194 (3)	
H6	0.0219	0.4232	0.8099	0.023*	
C7	0.0287 (2)	0.17919 (14)	0.66696 (13)	0.0153 (3)	
H7	0.0028	0.0869	0.6258	0.018*	
C8	0.1816 (2)	0.25341 (14)	0.64252 (13)	0.0160 (3)	
H8	0.2078	0.3484	0.6764	0.019*	
C9	0.3096 (2)	0.18722 (14)	0.56259 (13)	0.0142 (3)	
C10	0.6883 (2)	0.31779 (14)	0.41785 (13)	0.0149 (3)	
C11	0.8125 (2)	0.25900 (14)	0.33038 (13)	0.0151 (3)	
C12	0.9921 (2)	0.33116 (15)	0.32771 (14)	0.0180 (3)	
H12	1.0378	0.4122	0.3850	0.022*	
C13	1.1038 (2)	0.28350 (16)	0.24048 (15)	0.0220 (3)	
H13	1.2270	0.3312	0.2393	0.026*	
C14	1.0350 (2)	0.16644 (16)	0.15541 (15)	0.0241 (3)	
H14	1.1097	0.1358	0.0946	0.029*	
C15	0.8579 (2)	0.09389 (16)	0.15851 (15)	0.0248 (3)	
H15	0.8125	0.0130	0.1010	0.030*	
C16	0.7471 (2)	0.14018 (15)	0.24624 (14)	0.0197 (3)	
H16	0.6261	0.0904	0.2487	0.024*	

### Atomic displacement parameters (Å^2^)

**Table:**

	*U*^11^	*U*^22^	*U*^33^	*U*^12^	*U*^13^	*U*^23^
O1	0.0268 (6)	0.0377 (7)	0.0412 (7)	−0.0068 (5)	0.0216 (5)	−0.0036 (6)
O2	0.0277 (6)	0.0234 (6)	0.0190 (5)	0.0054 (4)	0.0076 (4)	−0.0003 (4)
O3	0.0205 (5)	0.0113 (5)	0.0255 (5)	0.0010 (4)	0.0099 (4)	0.0008 (4)
O4	0.0240 (5)	0.0109 (5)	0.0323 (6)	0.0005 (4)	0.0144 (5)	−0.0002 (4)
N1	0.0190 (6)	0.0233 (7)	0.0209 (6)	0.0034 (5)	0.0092 (5)	0.0024 (5)
N2	0.0185 (6)	0.0106 (6)	0.0209 (6)	0.0027 (5)	0.0125 (5)	0.0007 (5)
N3	0.0173 (6)	0.0114 (6)	0.0208 (6)	0.0021 (5)	0.0111 (5)	0.0007 (5)
C1	0.0144 (6)	0.0171 (7)	0.0145 (6)	0.0026 (5)	0.0040 (5)	0.0032 (5)
C2	0.0181 (7)	0.0159 (7)	0.0198 (7)	−0.0014 (5)	0.0059 (6)	−0.0013 (5)
C3	0.0169 (7)	0.0218 (7)	0.0239 (7)	−0.0040 (6)	0.0090 (6)	0.0005 (6)
C4	0.0147 (7)	0.0207 (7)	0.0169 (7)	0.0028 (5)	0.0069 (5)	0.0013 (6)
C5	0.0212 (7)	0.0151 (7)	0.0219 (7)	0.0009 (6)	0.0079 (6)	−0.0012 (6)
C6	0.0192 (7)	0.0166 (7)	0.0250 (7)	−0.0014 (5)	0.0107 (6)	0.0020 (6)
C7	0.0156 (7)	0.0149 (7)	0.0156 (7)	0.0021 (5)	0.0042 (5)	0.0013 (5)
C8	0.0182 (7)	0.0127 (7)	0.0185 (7)	0.0031 (5)	0.0069 (5)	0.0010 (5)
C9	0.0133 (6)	0.0142 (7)	0.0154 (6)	0.0008 (5)	0.0037 (5)	0.0023 (5)
C10	0.0148 (6)	0.0131 (7)	0.0173 (7)	0.0008 (5)	0.0046 (5)	0.0019 (5)
C11	0.0174 (7)	0.0139 (7)	0.0168 (6)	0.0039 (5)	0.0077 (5)	0.0048 (5)
C12	0.0176 (7)	0.0166 (7)	0.0210 (7)	0.0011 (5)	0.0059 (6)	0.0034 (6)
C13	0.0185 (7)	0.0237 (8)	0.0282 (8)	0.0039 (6)	0.0119 (6)	0.0088 (6)
C14	0.0312 (8)	0.0234 (8)	0.0244 (8)	0.0090 (6)	0.0182 (7)	0.0063 (6)
C15	0.0373 (9)	0.0172 (7)	0.0228 (7)	0.0019 (6)	0.0133 (7)	0.0003 (6)
C16	0.0232 (7)	0.0159 (7)	0.0223 (7)	−0.0008 (6)	0.0104 (6)	0.0022 (6)

### Geometric parameters (Å, °)

**Table:**

O1—N1	1.2309 (16)	C5—H5	0.9500
O2—N1	1.2282 (16)	C6—H6	0.9500
O3—C9	1.2392 (16)	C7—C8	1.3360 (19)
O4—C10	1.2346 (16)	C7—H7	0.9500
N1—C4	1.4695 (17)	C8—C9	1.4784 (18)
N2—C9	1.3469 (17)	C8—H8	0.9500
N2—N3	1.3914 (16)	C10—C11	1.4905 (18)
N2—H2N	0.879 (9)	C11—C16	1.3913 (19)
N3—C10	1.3490 (18)	C11—C12	1.399 (2)
N3—H3N	0.878 (9)	C12—C13	1.394 (2)
C1—C2	1.3974 (19)	C12—H12	0.9500
C1—C6	1.402 (2)	C13—C14	1.388 (2)
C1—C7	1.4717 (18)	C13—H13	0.9500
C2—C3	1.3884 (19)	C14—C15	1.387 (2)
C2—H2	0.9500	C14—H14	0.9500
C3—C4	1.379 (2)	C15—C16	1.391 (2)
C3—H3	0.9500	C15—H15	0.9500
C4—C5	1.386 (2)	C16—H16	0.9500
C5—C6	1.3834 (19)		
			
O2—N1—O1	123.49 (12)	C1—C7—H7	116.8
O2—N1—C4	118.41 (11)	C7—C8—C9	119.74 (13)
O1—N1—C4	118.09 (12)	C7—C8—H8	120.1
C9—N2—N3	118.46 (11)	C9—C8—H8	120.1
C9—N2—H2N	125.6 (11)	O3—C9—N2	121.75 (12)
N3—N2—H2N	114.0 (11)	O3—C9—C8	124.24 (12)
C10—N3—N2	117.73 (11)	N2—C9—C8	113.95 (12)
C10—N3—H3N	126.1 (11)	O4—C10—N3	121.47 (12)
N2—N3—H3N	114.1 (11)	O4—C10—C11	122.51 (12)
C2—C1—C6	118.84 (12)	N3—C10—C11	115.96 (12)
C2—C1—C7	118.38 (13)	C16—C11—C12	119.75 (13)
C6—C1—C7	122.74 (12)	C16—C11—C10	121.48 (12)
C3—C2—C1	120.74 (13)	C12—C11—C10	118.62 (13)
C3—C2—H2	119.6	C13—C12—C11	119.66 (14)
C1—C2—H2	119.6	C13—C12—H12	120.2
C4—C3—C2	118.63 (13)	C11—C12—H12	120.2
C4—C3—H3	120.7	C14—C13—C12	120.04 (14)
C2—C3—H3	120.7	C14—C13—H13	120.0
C3—C4—C5	122.36 (13)	C12—C13—H13	120.0
C3—C4—N1	118.74 (12)	C13—C14—C15	120.45 (13)
C5—C4—N1	118.87 (13)	C13—C14—H14	119.8
C6—C5—C4	118.45 (13)	C15—C14—H14	119.8
C6—C5—H5	120.8	C14—C15—C16	119.70 (14)
C4—C5—H5	120.8	C14—C15—H15	120.2
C5—C6—C1	120.90 (13)	C16—C15—H15	120.2
C5—C6—H6	119.5	C15—C16—C11	120.37 (13)
C1—C6—H6	119.5	C15—C16—H16	119.8
C8—C7—C1	126.49 (13)	C11—C16—H16	119.8
C8—C7—H7	116.8		
			
C9—N2—N3—C10	172.40 (12)	N3—N2—C9—O3	4.2 (2)
C6—C1—C2—C3	−2.4 (2)	N3—N2—C9—C8	−178.39 (11)
C7—C1—C2—C3	175.31 (13)	C7—C8—C9—O3	−9.0 (2)
C1—C2—C3—C4	0.3 (2)	C7—C8—C9—N2	173.72 (12)
C2—C3—C4—C5	2.4 (2)	N2—N3—C10—O4	0.0 (2)
C2—C3—C4—N1	−175.32 (13)	N2—N3—C10—C11	−177.37 (11)
O2—N1—C4—C3	161.15 (13)	O4—C10—C11—C16	−146.43 (14)
O1—N1—C4—C3	−17.9 (2)	N3—C10—C11—C16	30.87 (19)
O2—N1—C4—C5	−16.63 (19)	O4—C10—C11—C12	29.2 (2)
O1—N1—C4—C5	164.35 (13)	N3—C10—C11—C12	−153.50 (13)
C3—C4—C5—C6	−2.8 (2)	C16—C11—C12—C13	0.1 (2)
N1—C4—C5—C6	174.94 (12)	C10—C11—C12—C13	−175.56 (12)
C4—C5—C6—C1	0.5 (2)	C11—C12—C13—C14	1.1 (2)
C2—C1—C6—C5	2.0 (2)	C12—C13—C14—C15	−1.7 (2)
C7—C1—C6—C5	−175.60 (13)	C13—C14—C15—C16	1.0 (2)
C2—C1—C7—C8	−179.69 (13)	C14—C15—C16—C11	0.3 (2)
C6—C1—C7—C8	−2.1 (2)	C12—C11—C16—C15	−0.8 (2)
C1—C7—C8—C9	175.50 (12)	C10—C11—C16—C15	174.75 (13)

### Hydrogen-bond geometry (Å, °)

**Table:**

*D*—H···*A*	*D*—H	H···*A*	*D*···*A*	*D*—H···*A*
N2—H2n···O4	0.879 (10)	2.273 (14)	2.6470 (16)	105.5 (11)
N2—H2n···O4^i^	0.879 (10)	2.050 (11)	2.8721 (15)	155.4 (13)
N3—H3n···O3	0.878 (10)	2.354 (14)	2.6687 (15)	101.3 (10)
N3—H3n···O3^ii^	0.878 (10)	2.070 (11)	2.9269 (15)	165.0 (13)
C12—H12···O4^iii^	0.95	2.56	3.4257 (18)	151
C14—H14···O1^iv^	0.95	2.58	3.2851 (19)	132

Symmetry codes:  (i) −*x*+1, −*y*+1, −*z*+1; (ii) −*x*+1, −*y*, −*z*+1; (iii) −*x*+2, −*y*+1, −*z*+1; (iv) *x*+2, *y*, *z*−1.

## References

[bb1] Bezerra, D. P., Castro, F. O., Alves, A. P. N. N., Pessoa, C., Moraes, M. O., Silveira, E. R., Lima, M. A. S., Elmiro, F. J. M. & Costa-Lotufo, L. V. (2006). *Braz* *J* *Med* *Biol* *Res* **39**, 801-807. 10.1590/s0100-879x2006000600014 16751987

[bb2] Brandenburg, K. (2006). *DIAMOND* Crystal Impact GbR, Bonn, Germany.

[bb3] Carvalho, S. R., da Silva, E. F., de Souza, M. V. N., Lourenço, M. C. S. & Vicente, F. R. (2008). *Bioorg* *Med* *Chem* *Lett* **18**, 538–541. 10.1016/j.bmcl.2007.11.091 18068364

[bb4] Carvalho, S. A., da Silva, E. F., Tiekink, E. R. T., Wardell, J. L. & Wardell, S. M. S. V. (2009). *Acta Cryst.* E**65**, o3118. 10.1107/S1600536809048156 PMC2971955 21578843

[bb5] Chung, H. S. & Shin, J. C. (2007). *Food Chem.* **104**, 1670–1677.

[bb6] Farrugia, L. J. (1997). *J. Appl. Cryst.* **30**, 565.

[bb7] Hooft, R. W. W. (1998). *COLLECT* Nonius BV, Delft, The Netherlands.

[bb8] Naz, S., Ahmad, S., Rasool, S. A., Sayeed, S. A. & Siddiqi, R. (2006). *Microb* *Res* **161**, 43-48. 10.1016/j.micres.2005.05.001 16338589

[bb9] Otwinowski, Z. & Minor, W. (1997). *Methods in Enzymology*, Vol. 276, *Macromolecular Crystallography*, Part A, edited by C. W. Carter Jr & R. M. Sweet, pp. 307–326. New York: Academic Press.

[bb10] Sheldrick, G. M. (2007). *SADABS* Bruker AXS Inc., Madison, Wisconsin, USA.

[bb11] Sheldrick, G. M. (2008). *Acta Cryst.* A**64**, 112–122. 10.1107/S0108767307043930 18156677

[bb12] Westrip, S. P. (2009). *publCIF* In preparation.

